# Image fusion-based low-dose CBCT enhancement method for visualizing miniscrew insertion in the infrazygomatic crest

**DOI:** 10.1186/s12880-024-01289-2

**Published:** 2024-05-17

**Authors:** Peipei Sun, Jinghui Yang, Xue Tian, Guohua Yuan

**Affiliations:** 1 State Key Laboratory of Oral & Maxillofacial Reconstruction and Regeneration, Key Laboratory of Oral Biomedicine Ministry of Education, Hubei Key Laboratory of Stomatology, School & Hospital of Stomatology, Wuhan University, Wuhan, China; 2 Department of Pediatric Dentistry, School and Hospital of Stomatology, Wuhan University, Wuhan, China; 3 Frontier Science Center for Immunology and Metabolism, Wuhan University, Wuhan, China

**Keywords:** Low-dose CBCT, Infrazygomatic crest, Image processing, Miniscrew

## Abstract

Digital dental technology covers oral cone-beam computed tomography (CBCT) image processing and low-dose CBCT dental applications. A low-dose CBCT image enhancement method based on image fusion is proposed to address the need for subzygomatic small screw insertion. Specifically, firstly, a sharpening correction module is proposed, where the CBCT image is sharpened to compensate for the loss of details in the underexposed/over-exposed region. Secondly, a visibility restoration module based on type II fuzzy sets is designed, and a contrast enhancement module using curve transformation is designed. In addition to this, we propose a perceptual fusion module that fuses visibility and contrast of oral CBCT images. As a result, the problems of overexposure/underexposure, low visibility, and low contrast that occur in oral CBCT images can be effectively addressed with consistent interpretability. The proposed algorithm was analyzed in comparison experiments with a variety of algorithms, as well as ablation experiments. After analysis, compared with advanced enhancement algorithms, this algorithm achieved excellent results in low-dose CBCT enhancement and effective observation of subzygomatic small screw implantation. Compared with the best performing method, the evaluation metric is 0.07–2 higher on both datasets. The project can be found at: https://github.com/sunpeipei2024/low-dose-CBCT.

## Introduction

Low-dose CBCT oral image enhancement [1–4] is important in the field of dentistry and dentistry because it uses diagnostic examinations performed with low X-ray radiation doses to reduce the radiation exposure to the human body while improving the quality of the CBCT oral images and to provide a more accurate diagnosis and treatment plan for the patient’s oral disease. The following are some of the important implications of low-dose CBCT oral image enhancement: (1) Improvement of image quality [5]: low-dose CBCT is a technique for obtaining structural images of the oral cavity by X-ray scanning. By enhancing the images, the contrast and clarity of the images can be improved, helping the physician to better visualize oral structures, identify details and reduce noise. (2) Improve diagnostic accuracy [6]: oral image enhancement can help doctors diagnose problems with teeth and oral structures more accurately. (3) Reduced radiation dose [7]: An important advantage of low-dose CBCT is that it reduces the dose of X-ray radiation to which the patient is exposed. With image enhancement techniques, high quality images can be obtained at low radiation doses, thus reducing the potential risk of radiation to the patient.

CBCT is an important part of oral radiography and is now widely utilized in dental clinics due to its 3D high resolution at low cost [8]. For example, temporomandibular joint disorders, orthodontic treatment, and complex root canal treatment [9]. In orthodontic treatment, CBCT can provide accurate images of the hard tissues at the zygomatic alveolar ridge and infrazygomatic crest [10], in addition, as the only diagnostic imaging technique, it can roughly determine the structure and density of the jawbone, evaluate the anatomy of the cortical and cancellous bone, and give us a reference to add an assistive device in moving the posterior teeth [11]. However, CBCT is genotoxic and cytotoxic to oral mucosal cells not only in children but also in adults and may increase the shedding of human oral mucosal cells [12]. The risk of exposure to dental CBCT rays increases with dose. This radiation exposure may lead to tissue damage, especially to tissues of the head and neck [13]. Therefore, dentists should use CBCT scans with caution to minimize the radiation dose received by the patient and use lower dose scanning parameters whenever possible. In addition, patients should be informed of the potential risks and an adequate risk and benefit assessment should be performed before undergoing a CBCT scan [14]. CBCT images with stainless steel crowns implants or restorations will show streaks and shadows due to its imaging properties, which reduces the contrast of the image, and low-quality CBCT also introduces an additional error at the alignment stage, leading to a reduction in accuracy.

**Fig. 1:**
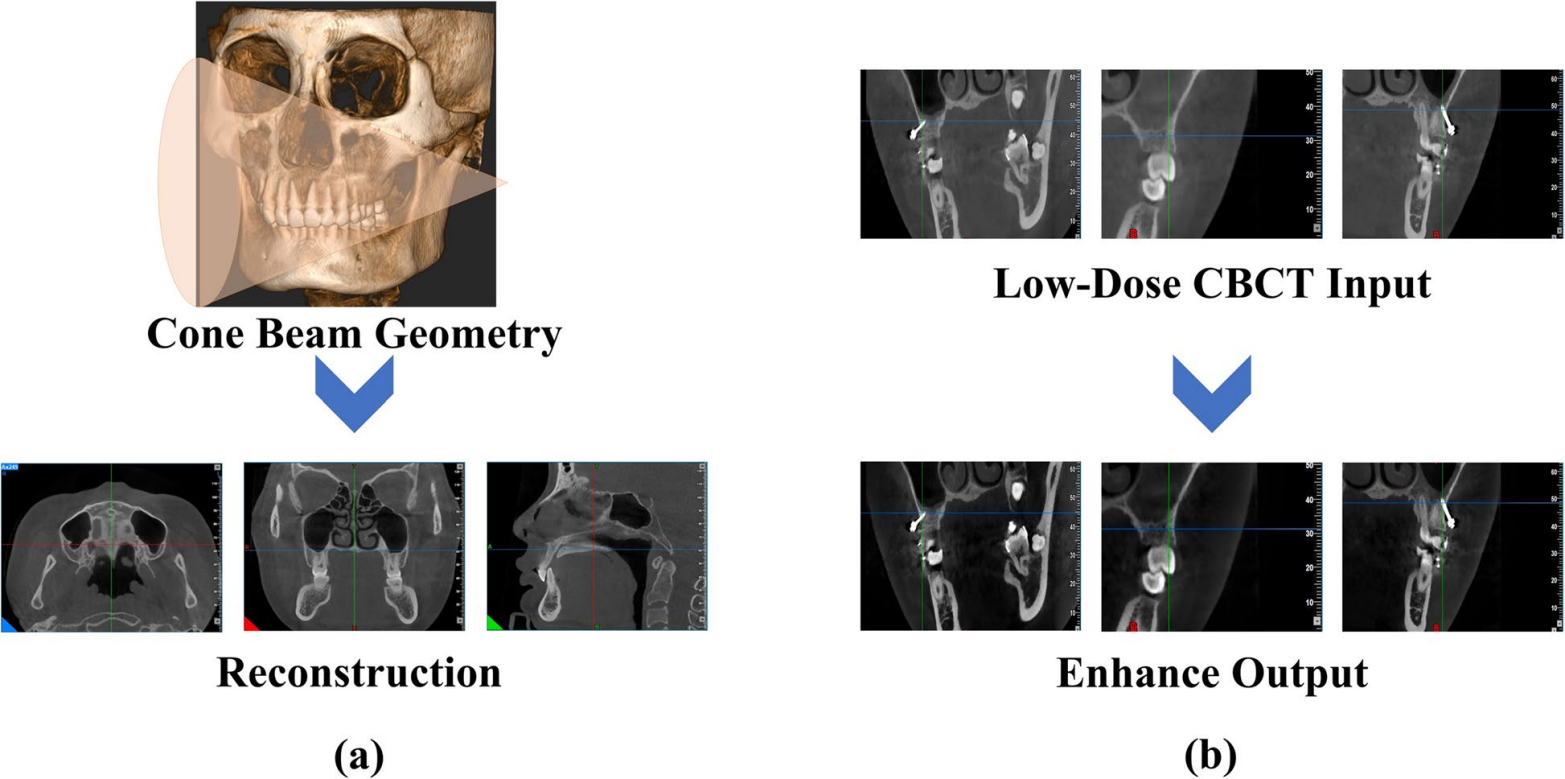
CBCT imaging and low-dose CBCT image enhancement effects. CBCT imaging does a great job of photographing the oral cavity. The proposed method proposes a solution to the problem of oral low-dose CBCT images. As shown in (**b**), the proposed method can well solve the problem of oral low-dose CBCT images

Bone class II high angle patients have overdeveloped posterior alveolar bone, often need to simulate Lefort type I surgery to depress the maxillary posterior teeth, and sometimes need the maximum support to retract the anterior teeth. Miniscrews meet these two requirements, the requirements of patient compliance is low, and the cost is less. However, the bone cortex of high-angle patients is thin, the alveolar bone thickness is relatively low, the infrazygomatic crest floor is low, and the miniscrews are easily loosened and dislodged. A large number of studies at home and abroad have been built on the study of CBCT, but because all kinds of miniscrews are basically made of stainless steel, it will have the problem of accuracy for CBCT images. In this paper, an in-depth study is conducted to improve the quality of CBCT images and to solve the problem of radiologic accuracy due to the low quality of CBCT images. Figure 1 shows CBCT imaging and the proposed method’s enhancement effect on low-dose CBCT images.

Each method has its advantages and disadvantages when dealing with low-dose CBCT oral image enhancement. For traditional methods, many articles only consider visibility or noise, but we believe that a combination of degradation factors is needed to make enhancement of low-dose CBCT images, which may be less practical for practical clinical applications. For deep learning methods, many articles usually require validation in the dental field. In addition to this, the complexity and black-box nature of deep learning models may raise some concerns in the clinical setting.

In this study, we take full advantage of mathematical transformations to address the problem of degradation in the quality of low-dose CBCT oral images by using an integrated approach to highlight the representation of different tasks. Unlike previous approaches that focus only on low visibility, we extensively consider the causes of degradation in low-dose CBCT oral images. First, we propose a sharpness enhancement method based on mathematical transformations to compensate for the loss of details in the exposed region. The method subtly enhances the sharpness of the image through mathematical transformations, thus improving the overall quality. Secondly, we designed a visibility restoration module based on type II fuzzy sets to deal with the problem of low-dose CBCT oral images more comprehensively. Meanwhile, we introduce a contrast enhancement module using curve transformation, which helps to improve the contrast in the image and makes the key features more prominent. In addition to this, we innovatively propose a perceptual fusion module that further improves oral CBCT images by fusing the information of visibility and contrast. This approach has rarely been studied in the field of low-dose CBCT oral image enhancement and provides new ideas and ways to solve related problems. It is worth mentioning that since our method is based on a non-physical model, it exhibits exciting characteristics in the representation of biological visual properties. Meanwhile, our method is highly interpretable, which enables us to clearly understand the contribution of each module to image enhancement, providing strong support for the tuning and improvement of the method. The main contributions of this paper are as follows:

- To solve the problem of detail loss in the underexposed/ overexposed regions of low-dose CBCT oral images, we propose a sharpening enhancement method based on mathematical transforms.
- To solve the problem of visibility and contrast degradation in low-dose CBCT oral images, we design a visibility restoration module based on type II fuzzy sets, and we design a contrast enhancement module using curve transformation.
- We propose a perceptual fusion strategy that simultaneously considers two images, fuses the visibility restored and contrast enhanced CBCT images, and considers both pixel intensity and global gradient changes in both images.

## Related works

In recent years, research for low-dose CBCT image enhancement has made significant progress in several areas. These researches mainly focus on two directions: traditional methods and deep learning methods. The studies of traditional methods [15–20] mainly rely on classical image processing techniques and mathematical operations to improve the quality and sharpness of CBCT images. These methods include various filtering techniques, edge enhancement, histogram equalization, etc. By pre-processing, denoising and enhancing the images, the traditional methods try to improve the visualization of CBCT images. However, traditional methods may suffer from performance limitations when dealing with complex problems, especially when dealing with images with complex structures and noise. On the other hand, deep learning methods [21–23] show great potential in CBCT image enhancement. These methods utilize deep learning models such as deep neural networks and convolutional neural networks to achieve automatic image restoration and enhancement by learning features and patterns from large amounts of data. Deep learning methods are able to learn more complex and abstract features from large amounts of data, and therefore usually achieve better results when dealing with complex problems in CBCT images.

Overall, there are advantages and disadvantages to both traditional and deep learning methods. Traditional methods, which rely on feature extraction designed by engineers and rules formulated by hand, have the advantage of being highly interpretable and computationally fast, but may be limited when dealing with complex problems. Deep learning methods [24–26], on the other hand, are able to learn complex feature representations from a large amount of data and have stronger generalization capabilities, but require a large amount of labeled data and computational resources. Currently, many image enhancement applications have been proposed, showing the potential role of stochastic resonance [27–31], underwater image enhancement [32], and dehazing [33] in enhancing contrast. In this section, we will introduce the application of the two methods on oral CBCT image enhancement, respectively.

### Traditional methods

Many of the techniques in traditional methods are based on mathematical operations. For example, common filters (e.g., median filters, Gaussian filters) can reduce noise in an image by averaging or weighted averaging local pixel values over the image. Hart et al. [15] determined the optimal parameters in the variable kernel deformation image alignment of CBCT images to improve the accuracy and convergence of on-line adaptive radiotherapy. Churchill et al. [17] combined image processing techniques and statistical reconstruction by using initial filtered inverse projection reconstruction to create binary edge masks, which were then used for weighted regularized reconstruction. Chen et al. [18] proposed a new physical model-based approach to enhance the contrast of CT images. The input image is converted into a tissue parameter map using the relationship between tissue parameters. By using a classical parameter fitting model, a partially attenuated image with enhanced image contrast can be computed. Soltanian-Zadeh et al. [19] utilized the frequency characteristics of artifacts to identify and correct artifacts. Villain et al. [20] enhanced CT images by applying a semi-quadratic edge-preserving image restoration (or inverse convolution). This method can be used with almost any CT scanner as long as the overall point spread function can be roughly estimated. These methods are usually less adaptable to different image conditions and specific problems, and may not be able to accommodate a wide range of dental images. While traditional methods do not require much investment in training data, they may suffer from performance limitations when dealing with complex problems. The performance of these methods depends on the feature extraction designed by engineers and the rules formulated manually, and thus may not be well adapted to the complex structure and noise in the image. Compared to deep learning methods, traditional methods are usually more interpretable and computationally efficient, but may exhibit limitations when dealing with complex problems.

### Deep learning methods

Deep learning methods have achieved excellent performance in image enhancement tasks to capture complex features and textures in images. Deep learning methods are highly adaptable and can be used for different types of dental images and various problems. Wang et al. [34] performed multiclass segmentation of jaws, teeth and background from CBCT scans. Fully automated segmentation method for simultaneous segmentation of two anatomical structures in CBCT scans. Kida et al. [35] proposed a method using deep neural network CBCT images in response to shorter time and fewer exposures for acquiring CBCT images. Madesta et al. [36] used a convolutional neural network architecture with residual dense networks for interpreting inter-motor variability of targets. Griner et al. [37] developed a deep learning method for scattering-induced artifacts that can significantly degrade image quality to empirically correct the most commonly observed artifacts in CBCT-based images. Deep learning methods typically require large amounts of label data for training, which can be challenging for some applications. Deep learning models are usually black-box and it is difficult to explain their inner workings. Deep learning models usually require large amounts of labeled data for training, especially in tasks that require learning complex features and patterns. For example, in an image enhancement task, if a deep learning model is to be trained to improve the quality of low-dose CBCT images, a large amount of paired data with corresponding high-quality images is required. Collecting and labeling these data may require significant time and human resources and may be impractical for some applications.

## Proposed method

**Fig. 2:**
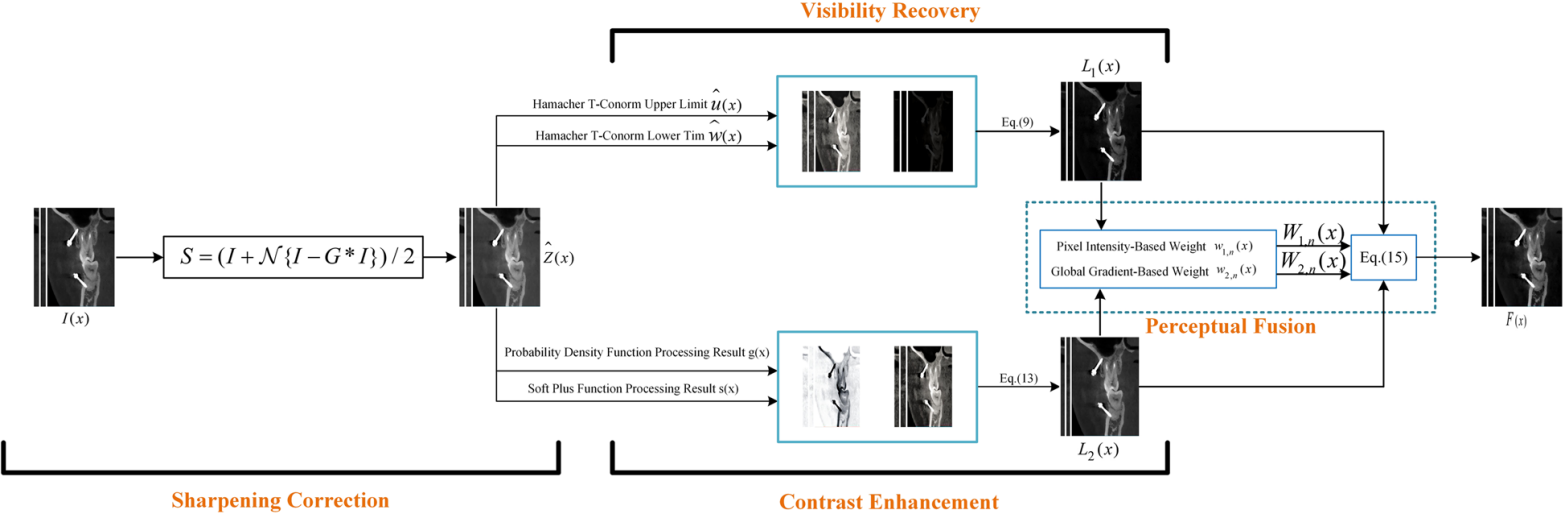
Overall framework. The input CBCT image is passed through sharpening correction, visibility restoration and contrast enhancement modules and finally fed into the perceptual fusion module for fusion

In this section, we illustrate the proposed method, which consists of four basic components. These four components include sharpening correction, visibility restoration, contrast enhancement and image fusion modules. The overall flowchart of the proposed method is shown in Fig. 2.

### Sharpening correction module

First, we input the CBCT image and sharpen the input oral CBCT image, whose main function is to enhance the edges and details of the image so that the image looks clearer and sharper. We define the processing result of the sharpened image $\hat Z$ by the following equation:

1 $$\hat Z = (I + \mathcal{N}\{ I - G*I\} )/2,$$

in Eq. (1), $G * I$ is defined as the Gaussian filtering result of $I$. $\mathcal{N}\{ \cdot \}$ is defined as the normalization operator. Oral CBCT images are sharpened to make the CBCT image clearer and sharper. It helps to highlight the differences between the boundaries and regions in the image. Sharpening the image can make the edges of the objects clearer and more visible, thus improving the visual quality of the image.

### Visibility recovery module

In the visibility restoration module, we work on the visual clarity enhancement of CBCT images. In order to realize the visual clarity recovery of CBCT images, we introduce an innovative method based on type II fuzzy sets. Based on the theory of type II fuzzy sets, we propose a new upper and lower range solution to the Hamacher t-conorm, and employ a transform-based gamma correction technique to accomplish the enhancement of CBCT image visibility. Through the combined application of these technological tools, we aim to improve the quality and clarity of CBCT images to more accurately support relevant applications and medical diagnosis. First, the mean $\mu$ and standard deviation $\sigma$ of the fuzzy image $\hat Z(x)$ are calculated:

2 $$\mu = \frac{1}{n} \cdot \sum\limits_{i = 1}^n {\hat Z({x_i})} ,$$

3 $$\sigma = \sqrt {\frac{1}{n - 1} \cdot \sum\limits_{i = 1}^n {{{\left( {\hat Z({x_i}) - \mu } \right)}^2}} } ,$$

based on Eqs. (2) and (3), we compute the new lower bound for the Hamacher t-conorm. Here, we compute the new upper bound $\hat u(x)$ by the following equation:

4 $$\hat u(x) = {\left( {\hat Z(x)} \right)^\alpha } + \left( {1 - {{\left( {\hat Z(x)} \right)}^\alpha }} \right) \cdot {\left( {\sigma^2} \right)^\alpha },$$

where $\alpha = 0.95$. The new lower limit $\hat w(x)$ is expressed using the following equation:

5 $$\hat w(x) = \left( {\frac{k \cdot \mu }{{\sigma + b}}} \right) \cdot \left( {\hat Z(x) - c \cdot \mu } \right) + {\mu^d}.$$

Hamacher t-conorm is an operation used in fuzzy logic to merge the membership values of two fuzzy sets. When calculating the new Hamacher t-conorm, we need to ensure that the updated lower and upper bounds are taken into account in order to accurately reflect the relationship between the fuzzy sets. This will ensure accurate results when dealing with fuzzy data, thus increasing the reliability of mathematical and statistical applications:

6 $$t(x) = \frac{{\hat u(x) + \hat w(x) + \left( {{\sigma^2} - 2} \right) \cdot \hat u(x) \cdot \hat w(x)}}{{1 - \left( {1 - {\sigma^2}} \right) \cdot \hat u(x) \cdot \hat w(x)}}.$$

Gamma correction can be used to improve the visual quality of CBCT images when they appear dull or unclear after processing. By remapping the pixel values of the input CBCT image, the sharpness and contrast of the image is enhanced. Gamma correction is based on a nonlinear transformation of the pixel values of an image using a gamma function. The gamma function adjusts the brightness and contrast of the image so that dark and bright details are more prominent:

7 $${L_1}(x) = \max \left( {t(x)} \right) \cdot {\left( {\frac{t(x)}{{\max \left( {t(x)} \right)}}} \right)^{1.5 \cdot \alpha }},$$

where ${L_1}(x)$ is the final output of the visibility restoration module.

### Contrast enhancement module

The main goal of the contrast enhancement module is to improve the contrast of CBCT images. To achieve this goal, we first process the image using two unique curve transformation functions to produce an image that is significantly enhanced in contrast by combining their outputs. Next, we introduce a gamma-corrected stretching function that stretches the intensity of the image to conform to standard intervals. The key to this process is to effectively enhance the grayscale differences in the image through the combined application of different transforms and adjustments, making the details in the image more prominent and legible, and providing a more reliable basis for subsequent medical image analysis and diagnosis. Combining Eqs. (8) and (9), we can apply the probability density function and the soft additive function of the standard normal distribution to each pixel value of the CBCT image in order to realize the individual processing of the image and to improve the visual quality of the image and the ability to express information:

8 $$g(x) = \frac{1}{{\sqrt {2\pi } }}\exp \left( { - \frac{{{{\left( {\hat Z(x)} \right)}^2}}}{2}} \right),$$

9 $$s(x) = \log \left( {1 + \exp \left( {\hat Z(x)} \right)} \right).$$

Then, the logarithmic image processing method using Eq. (10) combines the outputs of these two methods:

10 $$l(x) = \sqrt {f(x) + s(x) + f(x) * s(x)} .$$

Finally, a gamma-controlled normalization function is applied via Eq. (11) in order to fully stretch the image intensities to standard intervals:

11 $${L_2}(x) = {\left( {\frac{{l(x) - \min \left( {l(x)} \right)}}{{\max \left( {l(x)} \right) - \min \left( {l(x)} \right)}}} \right)^\eta }.$$

The results obtained through the contrast enhancement module have improved contrast while maintaining brightness and natural. Where $g(x)$ is the generated contrast modified image, $\hat Z(x)$ is the input contrast distorted image and ${L_2}(x)$ is the contrast stretched image by normalization. Where $\eta = 0.8$ is the gamma correction parameter responsible for adjusting the contrast of the image.

### Perceptual fusion module

In this section, we successfully obtain visibility restored CBCT images and contrast enhanced CBCT images. Unlike traditional image fusion methods, our image fusion method stems from two independent tasks. Therefore, we propose a novel fusion strategy that simultaneously considers the weight assignment of the two images. This weight assignment consists of two aspects: a weight based on pixel intensity and a weight based on global gradient. By integrating the pixel-level intensity information and the gradient characteristics of the overall image, our method is able to capture and utilize the beneficial information generated by the two different tasks more comprehensively during the image fusion process, thus further improving the quality and information content of the synthesized image. This innovative weight adjustment strategy injects higher flexibility and adaptability into our image fusion method, enabling it to perform well in different scenarios and tasks.

#### Weight design based on pixel intensity

The pixel intensity based fused image $F(x)$ as a weighted sum of images can be expressed as:

12 $$F(x) = {W_1}(x){L_1}(x) + {W_2}(x){L_2}(x),$$

where ${W_1}(x)$ and ${W_2}(x)$ denote the weights of the importance of pixels ${L_1}(x)$ and ${L_2}(x)$. Thus, $W(x)$ gives more weight to regions where the pixel intensities perform well, ${m_n}$ is denoted as the average of the pixel intensities, and the weight should be larger when ${L_n}(x)$ is close to $1 - {m_n}$, which can be denoted as $\exp \left( { - {{\left( {{L_n}(x) - \left( {1 - {m_n}} \right)} \right)}^2}} \right)$. When processing an image, it is important to take into account the exposure level of the input image. This is because a large difference between the brightness of the images results in more well-exposed pixels. To account for this, a larger value of ${\sigma_N}$ is assigned when there is a significant difference in the average brightness between images. The weights based on pixel intensity are denoted as:

13 $${w_{1,n}}(x) = \exp \left( { - \frac{{{{\left( {{L_n}(x) - \left( {1 - {m_n}} \right)} \right)}^2}}}{2\sigma_n^2}} \right).$$

Among them.

14 $${\sigma_n} = \left\{ {\begin{array}{*{20}{l}} {1.5\left( {{m_{n + 1}} - {m_n}} \right)}&{n = 1} \\ {0.75\left( {{m_{n + 1}} - {m_{n - 1}}} \right)}&{1 < n < N} \\ {1.5\left( {{m_n} - {m_{n - 1}}} \right)}&{n = N} \end{array}} \right.,$$

where $N$ is the number of images in a set of images. In Eq. (13), dark pixels are assigned a larger weight when ${m_n}$ is close to 1 and vice versa. In addition, when the average brightness is significantly different between images, the weights are assigned larger values.

#### Weight design based on global gradient

We observe that in regions lacking texture, images often have low contrast or small gradient values. Therefore, emphasizing only large gradient areas may not effectively highlight pixels within areas with smaller gradients. Based on this understanding, we introduce a global gradient weighting method aimed at emphasizing global contrast. In images with higher contrast, the cumulative histogram has smaller gradient values. Therefore, we need to give greater weight to pixels when they lie within the range of the cumulative histogram with relatively small gradients. In other words, we need to dynamically adjust the weight of each pixel based on its position and gradient information in the image. In areas with smaller gradients, we would expect the pixels to contribute more, as these tend to be areas of the image that lack texture. Therefore, we design a global gradient weighting method to better consider the global contrast when processing images and dynamically adjust the weight of pixels according to the gradient distribution of the image. This global gradient-based weight adjustment strategy makes our image fusion method more intelligent and comprehensive, able to adapt more flexibly under different image characteristics and contrast conditions, and effectively improve the overall image quality and information transfer. The weights based on global gradient can be expressed by the following equation:

15 $$w_{2,n}(x)=\frac{\operatorname{Grad}_n\left(L_n(x)\right)^{-1}}{\sum\limits_{n=1}^N\operatorname{Grad}_n\left(L_n(x)\right)^{-1}+\epsilon},$$

where $\epsilon$ is a very small positive value and ${\operatorname{Grad}_n}\left( {{L_n}(x)} \right)$ denotes the gradient of the cumulative histogram when the intensity is ${L_n}(x)$. In image processing, global gradient refers to the ability to perform a more comprehensive analysis of a CBCT image while taking into account the overall characteristics of the image. While traditional local gradient methods focus on local variations around specific pixels, global gradient methods capture a wider range of image contextual information by considering the gradients of distant pixels. This approach contributes to a better understanding of the structure and features of the entire image, thus improving the analysis of CBCT images. To calculate the final weights for each CBCT image, the two weights are combined and normalized using a specific equation:

16 $$W_n(x)=\frac{w_{1,n}(x)\times w_{2,n}(x)}{\sum\limits_{n=1}^Nw_{1,n}(x)\times w_{2,n}(x)+\epsilon}.$$

Using the weights obtained by Eq. (16), we can fuse the images according to Eq. (12).

## Results

In this section, we will discuss in depth the methods used for evaluation to get a full picture of their performance benefits. We are committed to a comprehensive evaluation of the proposed methods to ensure their validity and reliability in practical applications. To achieve this goal, we employ a variety of evaluation methods, including quantitative metrics and qualitative analysis, to assess the performance of the methods from different perspectives. In addition to the quantitative metrics, we will also perform a qualitative analysis to visually assess the processed images to visually observe the image quality, detail retention, and other aspects of the evaluation. This evaluation method can provide intuition and help us understand how the method performs in real scenes. To verify the effectiveness of each module, we also conducted a series of ablation experiments. By testing each module independently, we can gain insight into its impact on the final results and determine the performance contribution of each module. This step-by-step validation approach helps to ensure that each component of the methodology works effectively and ultimately yields high-quality results. We chose MATLAB 2022a as the tool that would support us in performing various image processing operations and experiments. The data for this article can be found at: https://osf.io/f9r8v/.

### Experiment settings

In this section, we provide a thorough description of the experimental setup, specifically, we applied four reference-free evaluation metrics to fully validate two reference-free image datasets. We comprehensively compare nine of the most representative and state-of-the-art image enhancement methods. First, we used four reference-free evaluation metrics to ensure a comprehensive assessment of image enhancement methods. These metrics consider the performance of image quality, contrast, and detail retention to provide comprehensive information for the experimental results. We chose two datasets with no reference images so that the performance of various image enhancement methods can be verified more comprehensively. It helps to evaluate the generalization ability of the methods. In this experiment, we compare nine of the most representative and state-of-the-art image enhancement methods. We choose these methods based on their wide application and sophistication in the literature to ensure that our comparison is representative. By using multiple evaluation metrics on different reference-free image datasets, we were able to gain a comprehensive understanding of the performance of each image enhancement method, providing a solid foundation for further analysis and conclusions. Such an experimental design and detailed validation process help to ensure our objective evaluation of the performance of image enhancement methods.

#### Compared methods

Nine image enhancement methods including CEDN [38], NLM [39], NNC [40], MID [41], BCD [42], GM [43], DCFD [44], DPRN [45], and SDCN [46] were compared on Test 1 and Test 2 low-dose CBCT oral miniscrew image datasets.

#### No-reference image quality assessment metrics

No-reference image quality assessment metrics means that reference information is not available for predicting image quality. When confronted with oral CBCT, we can only obtain the current oral CBCT image and evaluate the enhanced image. In this paper, we use the following no-reference image evaluation metrics as the basis: Brisque [47], natural image quality evaluator (NIQE) [48], FADE [49], average gradient (AG) [50]. The lower the Brisque [47] score, the higher the filtered image’s The lower the Brisque [47] score, the higher the fidelity of the filtered image and the less detail information is lost. The lower the NIQE [48] score, the more natural the image performance. The lower the FADE [49] score, the lower the density of fog. The higher the AG [50] score, the more detail and the higher the clarity of the image.

### Qualitative and quantitative comparisons on the Test 1 dataset

#### Qualitative comparisons

**Fig. 3:**
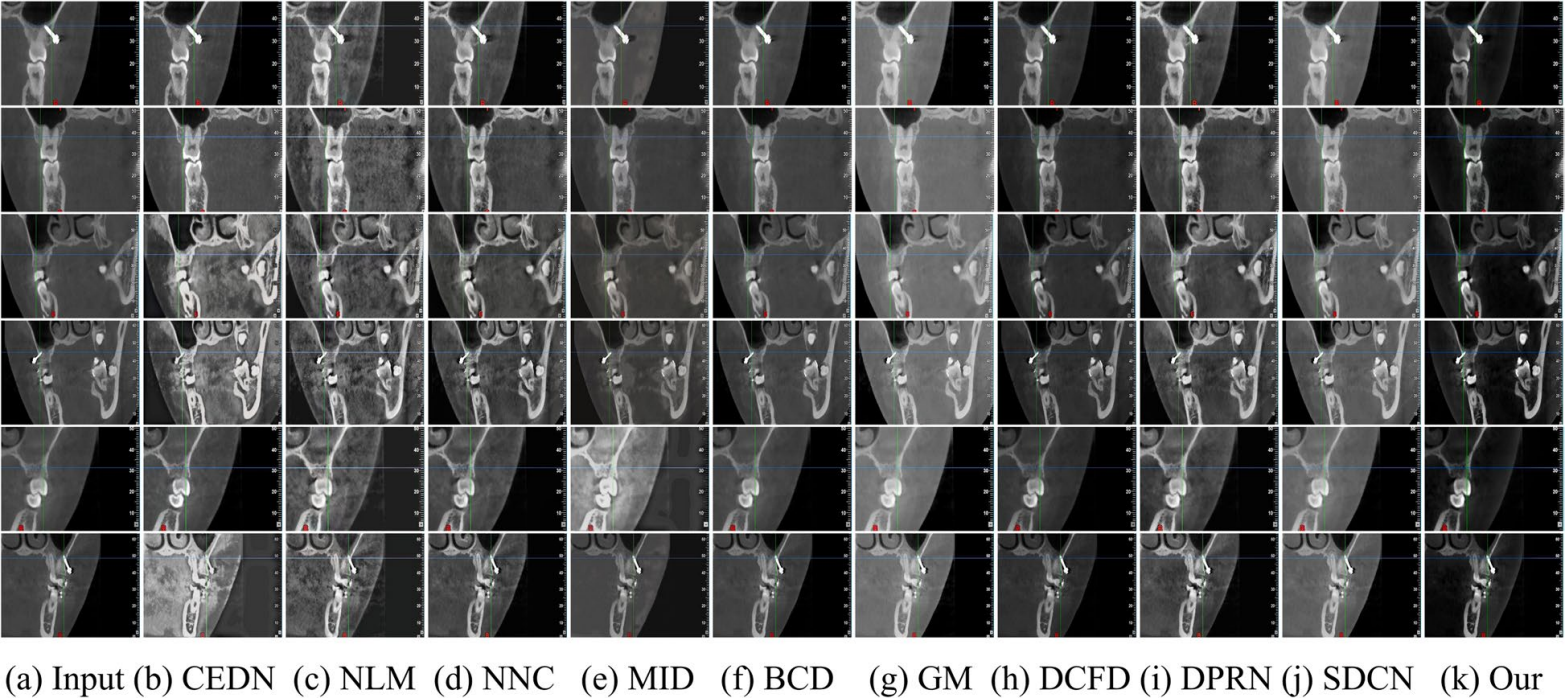
Comparative results of qualitative evaluation of different algorithms in Test 1 dataset. Here we show the enhancement effect of different algorithms on oral CBCT images

First, we compared the different methods in the Test1 dataset. As shown in Fig. 3, the CBCT image after CEDN [38] processing introduces new noise and exhibits an obvious white tone bias. The NLM-corrected image [39] shows more blocky areas and important details are not sufficiently highlighted, while producing obvious distortion. MID [41] improves the contrast of the panoramic image, but the correction effect is not obvious enough, and the process may lead to halo-like artifacts. NNC [40] Although the contrast was corrected to a certain extent, the overall image visibility was low. BCD [42] introduced a haze-like situation, which affected the image to a certain extent. GM [43] led to color deviation and local noise in the image. DCFD [44] failed to highlight local details in the overall image. DPRN [45] introduced unwanted white and grey noise during the correction process. The overall whiteness characteristic of the SDCN-enhanced image [46] and the enhancement information is not clear enough. In contrast, our method enhances the details of the CBCT image to the maximum extent, highlights the local contrast of the image, and successfully avoids the white balance distortion problem.

#### Quantitative comparisons

In order to comprehensively assess the differences in the performance of CBCT oral miniscrew images in terms of contrast, white balance, and visibility correction, we employed a variety of quantitative scoring metrics for in-depth analysis. Specifically, we utilized metrics such as Brisque [47], NIQE [48], FADE [49], and AG [50] scores to quantitatively assess the performance of different methods on the Test 1 dataset, and the relevant results are shown in Table 1. In the analysis of the Test 1 dataset, our method consistently performs well on all evaluation metrics, significantly outperforming the comparison methods. This demonstrates the superior performance of our method in terms of contrast, white balance and visibility correction. Considering all metrics together, our results clearly demonstrate the importance of robust image processing techniques to enhance the understanding of the oral miniscrew environment. These results not only emphasize the superiority of our method, but also the critical role of image processing in CBCT oral image analysis.

**Table 1:** The results of the unreferenced evaluation metrics compared to the nine algorithms are in Test 1

Methods	Test 1			
	Brisque↓	NIQE↓	FADE↓	AG↑
CEDN	70.6131	8.2567	1.1123	2.8567
NLM	72.3456	7.8976	1.0376	3.1590
NNC	69.1234	7.6234	1.1456	3.0234
MID	70.4321	8.7654	1.1832	2.9812
BCD	68.9876	8.4321	1.0598	3.1123
GM	78.1234	7.5432	1.1987	3.0456
DCFD	71.2345	8.3210	1.0054	2.8976
DPRN	79.8765	8.1234	1.0234	3.0654
SDCN	70.3615	7.9876	1.1256	3.1987
Our	67.6204	7.4832	0.9624	3.3588

### Qualitative and quantitative comparisons on the Test 2 dataset

#### Qualitative comparisons

**Fig. 4:**
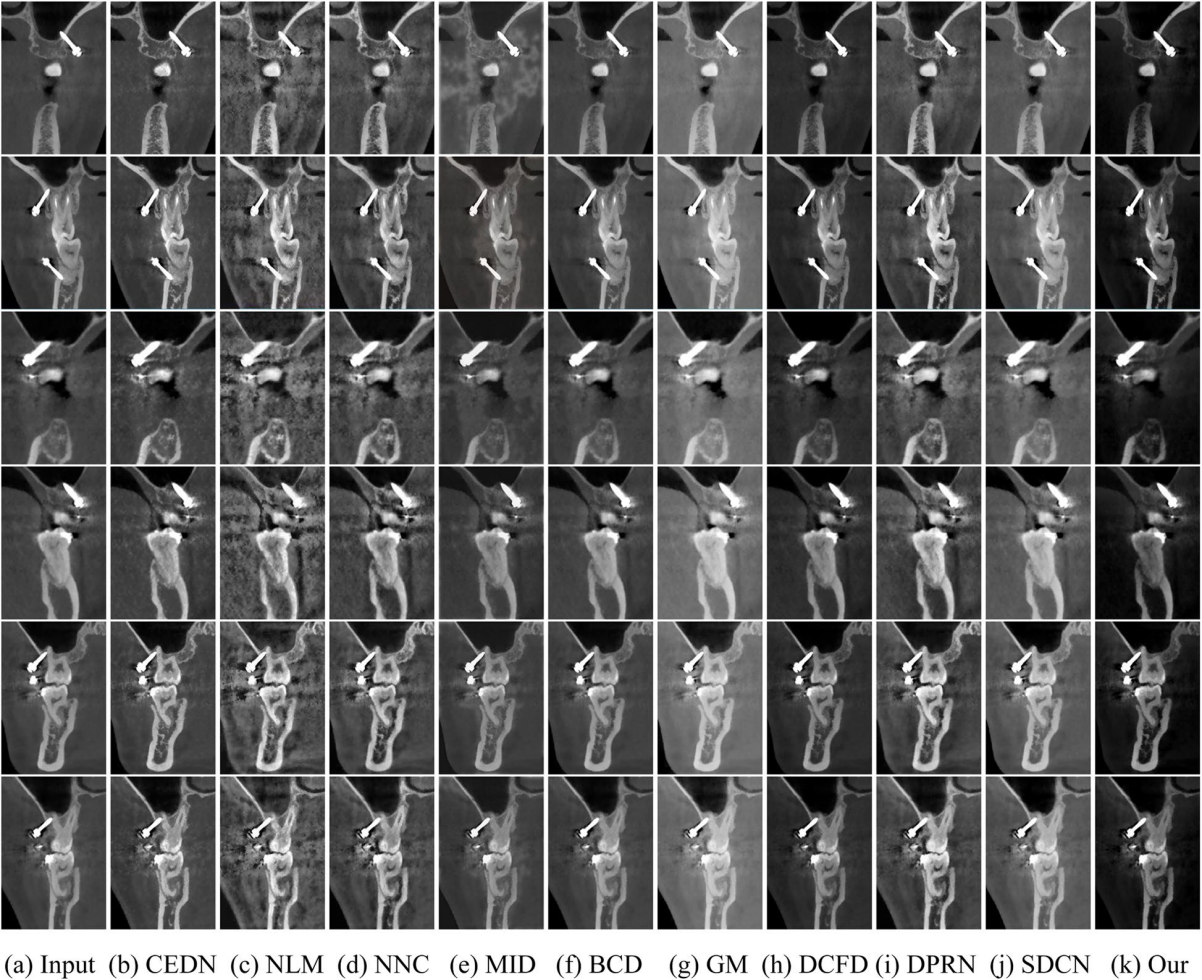
Comparative results of qualitative evaluation of different algorithms in Test 2 dataset. Here we show the enhancement effect of different algorithms on oral CBCT images

First, we compared the different methods on the Test2 dataset. As shown in Fig. 4, the CEDN [38] -processed image shows an overall noise phenomenon, especially metal artifacts are more obvious, which will significantly reduce the accuracy of the detection of the arch and arch thickness. The NLM [39] method introduces new noise, which makes the overall distribution become uneven. Although the NNC [40] method enhances the details of the image to a certain extent, it also introduces some noise. MID [41] retains the details of the image but introduces some foggy information. BCD [42] method does not show significant effect in image enhancement and cannot overcome the effect of the metal artifacts through post-processing. GM [43] method introduces white tones but weakens the expression of the detail information. MID [41] method introduces white tones but weakens the expression of the detail information. The DCFD [44] method was able to enhance the image contrast relatively stably, but still failed to highlight the details of the image. The DPRN [45] method enhanced the image contrast with white balance distortion, especially when the halo artifact phenomenon became obvious when the regions in the image were brighter than the surrounding regions. The halo phenomenon is manifested in the image as the edge portions of the highlighted regions show edges with lower brightness. On the contrary, the SDCN [46] method performs well in presenting detailed information in the image, but the visibility of the image is low. Comparatively, our method maximizes the details of the CBCT image while successfully avoiding the problem of different white balance distortions.

#### Quantitative comparisons

In order to comprehensively assess the performance of different methods on CBCT oral miniscrew images in terms of contrast, white balance, and visibility correction, we used a variety of scoring metrics for quantitative analysis. Specifically, we use four evaluation metrics: Brisque [47], NIQE [48], FADE [49], and AG [50]. We are able to clearly evaluate the performance of different algorithms through the results on the Test 2 dataset, and the detailed results are listed in Table 2. The experimental results on the Test 2 dataset show that our method outperforms the comparison algorithms on all four evaluation metrics, which suggests that the proposed algorithm is very good at visibility and white balance correction. In conclusion, by quantitatively comparing the proposed algorithm with several algorithms, we demonstrate the advantages of the proposed algorithm and analyze the importance of understanding CBCT images of small oral screws.

**Table 2:** The results of the unreferenced evaluation metrics compared to the nine algorithms are in Test 2

Methods	Test 2			
	Brisque↓	NIQE↓	FADE↓	AG↑
CEDN	51.2345	7.1234	1.7856	1.8790
NLM	54.5678	7.7890	1.5234	1.5432
NNC	52.1234	8.2345	1.9378	1.9876
MID	50.9876	6.9876	2.0456	1.3210
BCD	53.4321	7.4567	1.6543	2.0345
GM	51.8765	8.0987	1.8567	1.6543
DCFD	54.3210	6.8765	1.7890	1.7890
DPRN	50.3456	8.3456	2.0765	1.4321
SDCN	52.7890	7.5432	1.4321	1.9987
Our	49.6822	6.6040	1.3184	2.2253

### Ablation experiment

In this section, two image datasets are selected to ensure the generalizability of the experimental results. Select the image processing algorithms benchmarked by the proposed method for comparing the effectiveness of other processing methods. Apply the designed processing scheme to the selected image datasets to generate the processed images. Evaluate the processed images using the selected evaluation metrics and record the results. Ensure that sufficient sample size and statistical analysis methods are used in the experiment to draw reliable conclusions. We performed a quantitative evaluation through ablation experiments, the relevant results of which are listed in Table 3. Through these experiments, we gained a deeper understanding of each component’s contribution to improving the algorithm’s performance: 1) our method was performed without the visibility restoration module (-w/o VRM). 2) our method was performed without the contrast enhancement module (-w/o CEM). We evaluated and analyzed the effectiveness of this technique by means of an oral CBCT low-dose enhancement ablation experiment. The results of the oral CBCT low-dose enhancement ablation experiments demonstrated significant effectiveness in improving the quality of oral CBCT images and visualization of anatomical structures.

**Table 3:** Results of ablation experiments on two datasets

Ablated models	Test 1				Test 2			
	Brisque↓	NIQE↓	FADE↓	AG↑	Brisque↓	NIQE↓	FADE↓	AG↑
-w/o VRM	68.170	7.501	1.131	3.190	50.181	6.938	1.591	2.140
-w/o CEM	69.601	7.638	1.058	2.938	51.308	6.829	1.641	1.904
Full model	67.620	7.483	0.962	3.218	49.682	6.604	1.432	2.225

## Discussion

The proposed method takes into account the characteristics of oral low-dose CBCT images, overcomes the artifacts, detail loss, and color distortion caused by excessive enhancement of oral low-dose CBCT images, and maximizes the enhancement of oral low-dose CBCT images while retaining details and structural information. Judging from the evaluation index results of the two data sets, the proposed method has excellent results, but the method in this paper still has limitations and challenges. In order to improve the quality of low-dose CBCT images, more complex image reconstruction algorithms are necessary, such as iterative reconstruction techniques. However, these algorithms are computationally intensive and have high requirements on hardware equipment, which may increase costs and processing time. In addition, the trade-off between image noise and resolution is very important. While reducing radiation dose, the noise level of the image will often increase, which will affect the image quality. Improving noise control techniques often sacrifices some spatial resolution, an important consideration in medical applications that require high-precision diagnostics. In the future, we will work on solving trade-off issues like noise and resolution, and solving this problem based on deep learning is the focus of our research.

## Conclusion

In this paper, we propose an enhancement method for low-dose CBCT oral images based on image fusion. Specifically, we consider the low-dose CBCT image over/underexposure, visibility and contrast issues. In order to compensate for the loss of detail in the under/over-exposed regions, a sharpening correction module is proposed. A visibility restoration module based on type-II fuzzy sets is designed, and a contrast enhancement module using curve transformation is designed. In addition to this, we propose a perceptual fusion module that fuses visibility and contrast of oral CBCT images.

In this experiment, we conducted a detailed study for oral CBCT low-dose enhancement. By comparing the experimental results, compared with such enhancement methods, our method can effectively reduce the noise and artifacts in the image and improve the clarity and contrast of the image. It is able to observe the implantation of small subzygomatic screws more accurately. The details and contours of the image are clearer, which helps doctors to make accurate diagnosis and treatment planning. Our method maintains consistent interpretability, allowing physicians to understand the process of image enhancement and trust the results. Based on the results of our current study, we will continue to explore the following aspects to further improve the oral CBCT low-dose enhancement technique. We will further optimize the deep learning model to improve the enhancement of low-dose CBCT images.

## Data Availability

The data for this article can be found at: https://osf.io/f9r8v/. The project can be found at: https://github.com/sunpeipei2024/low-dose-CBCT.

## References

[1] Vijayan S, Luo MJ, Wu S (2022). Image enhancement of ultra-low dose CBCT images using a deep generative model. Oral Surg Oral Med Oral Pathol Oral Radiol.

[2] Matenine D, Schmittbuhl M, Bedwani S (2019). Iterative reconstruction for image enhancement and dose reduction in diagnostic cone beam CT imaging. J Xray Sci Technol.

[3] Ihlis RL, Kadesjö N, Tsilingaridis G, Benchimol D, Shi XQ. Image quality assessment of low-dose protocols in cone beam computed tomography of the anterior maxilla. Oral Surg Oral Med Oral Pathol Oral Radiol. 2022;133(4):483-91. 10.1016/j.oooo.2021.10.001 34742681

[4] Tsiklakis K, Donta C, Gavala S (2005). Dose reduction in maxillofacial imaging using low dose Cone Beam CT. Eur J Radiol.

[5] Hyun CM, Bayaraa T, Yun HS, Jang TJ, Park HS, Seo JK. Deep learning method for reducing metal artifacts in dental cone-beam CT using supplementary information from intra-oral scan. Phys Med Biol. 2022;67(17). 10.1088/1361-6560/ac8852 35944531

[6] van Bunningen RH, Dijkstra PU, Dieters A, van der Meer WJ, Kuijpers-Jagtman AM, Ren Y. Precision of orthodontic cephalometric measurements on ultra low dose-low dose CBCT reconstructed cephalograms. Clin Oral Investig. 2022;26(2):1543–50. 10.1007/s00784-021-04127-9. 10.1007/s00784-021-04127-9 PMC8816531 34453209

[7] Hao J, Zhang L, Li L (2012). A comparison of projection domain noise reduction methods in low-dose dental CBCT. 2012 IEEE Nuclear Science Symposium and Medical Imaging Conference Record (NSS/MIC).

[8] Altoukhi DH, Alaki S, El Ashiry E, Nassif O, Sabbahi D. Genotoxicity and cytotoxicity of cone beam computed tomography in children. BMC Oral Health. 2021;21(1):427. 10.1186/s12903-021-01792-w. 10.1186/s12903-021-01792-w PMC8418710 34481467

[9] Kobayashi K, Shimoda S, Nakagawa Y, Yamamoto A (2004). Accuracy in measurement of distance using limited cone-beam computerized tomography. Int J Oral Maxillofac Implants.

[10] Memon A, Rogers I, Paudyal P, Sundin J (2019). Dental X-rays and the risk of thyroid cancer and meningioma: a systematic review and meta-analysis of current epidemiological evidence. Thyroid.

[11] Quirynen M, Mraiwa N, van Steenberghe D, Jacobs R (2003). Morphology and dimensions of the mandibular jaw bone in the interforaminal region in patients requiring implants in the distal areas. Clin Oral Implants Res.

[12] Venkatesh E, Elluru SV (2017). Cone beam computed tomography: basics and applications in dentistry. J Istanb Univ Fac Dent.

[13] White SC (2008). Cone-beam imaging in dentistry. Health Phys.

[14] Yang P, Xuan B, Li G, Qi S (2023). Does cone-beam computed tomography examination increase the micronuclei frequency in the oral mucosa exfoliated cells? A systematic review and meta-analysis. BMC Oral Health.

[15] Hart V, Burrow D, Li XA (2017). A graphical approach to optimizing variable-kernel smoothing parameters for improved deformable registration of CT and cone beam CT images. Phys Med Biol.

[16] Reaungamornrat S, Wang AS, Uneri A (2014). Deformable image registration with local rigidity constraints for cone-beam CT-guided spine surgery. Phys Med Biol.

[17] Churchill V, Gelb A (2019). Edge-masked CT image reconstruction from limited data. In: 15th International Meeting on Fully Three-Dimensional Image Reconstruction in Radiology and Nuclear Medicine. SPIE.

[18] Chen YW, Shih CT, Lin HH (2016). Physical model-based contrast enhancement of computed tomography images: contrast enhancement of computed tomography. 2016 IEEE 16th International Conference on Bioinformatics and Bioengineering (BIBE).

[19] Soltanian-Zadeh H, Windham JP, Soltanianzadeh J (1996). CT artifact correction: an image-processing approach. Medical imaging 1996: image processing.

[20] Villain N, Goussard Y, Idier J (2003). Three-dimensional edge-preserving image enhancement for computed tomography. IEEE Trans Med Imaging.

[21] Lei Y, Wang T, Harms J (2019). Image quality improvement in cone-beam CT using deep learning. Medical imaging 2019: physics of medical imaging.

[22] Jiang Z, Chen Y, Zhang Y (2019). Augmentation of CBCT reconstructed from under-sampled projections using deep learning. IEEE Trans Med Imaging.

[23] Zhang Y, Yue N, Su MY (2021). Improving CBCT quality to CT level using deep learning with generative adversarial network. Med Phys.

[24] Ren Z, Kong X, Zhang Y, et al. UKSSL: underlying knowledge based semi-supervised learning for medical image classification. IEEE Open J Eng Med Biol. 2023;1–8. 10.1109/OJEMB.2023.3305190 PMC11186655 38899016

[25] Zhang Y, Deng L, Zhu H (2023). Deep learning in food category recognition. Inform Fusion.

[26] Ren Z, Wang S, Zhang Y (2023). Weakly supervised machine learning. CAAI Trans Intell Technol.

[27] Mohanty S, Dakua SP (2022). Toward computing cross-modality symmetric non-rigid medical image registration. IEEE Access.

[28] Regaya Y, Amira A, Dakua SP (2023). Development of a cerebral aneurysm segmentation method to prevent sentinel hemorrhage. Netw Model Anal Health Inform Bioinform.

[29] Dakua SP, Abinahed J, Al-Ansari A (2016). Pathological liver segmentation using stochastic resonance and cellular automata. J Vis Commun Image Represent.

[30] Dakua SP (2015). LV segmentation using stochastic resonance and evolutionary cellular automata. Int J Pattern Recognit Artif Intell.

[31] Dakua SP, Abinahed J, Al-Ansari A (2017). Cellular automata-based left ventricle reconstruction from magnetic resonance images. Comput Methods Biomech Biomed Eng Imaging Vis.

[32] An S, Xu L, Senior Member I (2024). HFM: a hybrid fusion method for underwater image enhancement. Eng Appl Artif Intell.

[33] An S, Huang X, Wang L (2022). Semi-supervised image dehazing network. Vis Comput.

[34] Wang H, Minnema J, Batenburg KJ (2021). Multiclass CBCT image segmentation for orthodontics with deep learning. J Dent Res.

[35] Kida S, Kaji S, Nawa K (2020). Visual enhancement of cone-beam CT by use of CycleGAN. Med Phys.

[36] Madesta F, Sentker T, Gauer T (2020). Self-contained deep learning-based boosting of 4D cone-beam CT reconstruction. Med Phys.

[37] Griner D, Garrett JW, Li Y (2020). Correction for cone beam CT image artifacts via a deep learning method. medical imaging 2020: physics of medical imaging.

[38] Shan H, Zhang Y, Yang Q (2018). 3-D convolutional encoder-decoder network for low-dose CT via transfer learning from a 2-D trained network. IEEE Trans Med Imaging.

[39] Green M, Marom EM, Kiryati N (2016). Efficient low-dose CT denoising by locally-consistent non-local means (LC-NLM). Medical Image Computing and Computer-Assisted Intervention-MICCAI 2016: 19th International Conference, Athens, Greece, October 17-21, 2016, Proceedings, Part III 19.

[40] Suzuki K, Liu J, Zarshenas A (2017). Neural network convolution (nnc) for converting ultra-low-dose to “virtual” high-dose ct images. Machine Learning in Medical Imaging: 8th International Workshop, MLMI 2017, Held in Conjunction with MICCAI 2017, Quebec City, QC, Canada, September 10, 2017, Proceedings 8.

[41] Wu D, Gong K, Kim K (2019). Consensus neural network for medical imaging denoising with only noisy training samples. International Conference on Medical Image Computing and Computer-Assisted Intervention.

[42] Chun IY, Zheng X, Long Y (2019). BCD-Net for low-dose CT reconstruction: acceleration, convergence, and generalization. Medical Image Computing and Computer Assisted Intervention–MICCAI 2019: 22nd International Conference, Shenzhen, China, October 13–17, 2019, Proceedings, Part VI 22.

[43] Zhang R, Ye DH, Pal D (2016). A Gaussian mixture MRF for model-based iterative reconstruction with applications to low-dose X-ray CT. IEEE Trans Comput Imaging.

[44] Kang E, Chang W, Yoo J (2018). Deep convolutional framelet denosing for low-dose CT via wavelet residual network. IEEE Trans Med Imaging.

[45] Yin X, Zhao Q, Liu J (2019). Domain progressive 3D residual convolution network to improve low-dose CT imaging. IEEE Trans Med Imaging.

[46] Liu P, Fang R (2018). SDCNet: Smoothed dense-convolution network for restoring low-dose cerebral CT perfusion. 2018 IEEE 15th International Symposium on Biomedical Imaging (ISBI 2018).

[47] Mittal A, Moorthy AK, Bovik AC (2012). No-reference image quality assessment in the spatial domain. IEEE Trans Image Process.

[48] Mittal A, Soundararajan R, Bovik AC (2012). Making a “completely blind” image quality analyzer. IEEE Signal Process Lett.

[49] Choi LK, You J, Bovik AC (2015). Referenceless prediction of perceptual fog density and perceptual image defogging. IEEE Trans Image Process.

[50] Liu R, Ma L, Zhang J, Fan X, Luo Z (2021). Retinex-inspired unrolling with cooperative prior architecture search for low-light image enhancement. Proc. IEEE/CVF Conf. Comput. Vis. Pattern Recognit. (CVPR).

